# Assessment of Microplastic and Element Contamination in Fish Sold by Informal Roadside Vendors in Limpopo Province, South Africa

**DOI:** 10.1007/s00244-026-01223-3

**Published:** 2026-10-26

**Authors:** Alice R. Tshifura, Nombuso N. Themba, Franco Teixeira-de-Mello, Linton F. Munyai, Farai Dondofema, Ncumisa Y. Matam, Tatenda Dalu

**Affiliations:** 1 Aquatic Systems Research Group, School of Biology and Environmental Sciences, University of Mpumalanga, Nelspruit, South Africa; 2 Centre for Invasion Biology, School for Climate Studies, Stellenbosch University, Stellenbosch, South Africa; 3 Departamento de Ecología y Gestión Ambiental, Centro Universitario Regional Este, Universidad de la República–Uruguay, Tacuarembó s/n, entre Av. Artigas y Aparicio Saravia, Maldonado, CP 20000 Uruguay; 4 Aquatic Systems Research Group, Department of Water and Sanitation, University of Limpopo, Sovenga, South Africa; 5 Aquatic Systems Research Group, Department of Geography and Environmental Sciences, University of Venda, Thohoyandou, South Africa

## Abstract

Fish is the cheapest source of protein, minerals, and vitamins for most households in sub–Saharan Africa. Of late, microplastic and metal pollution have raised concerns about food safety, especially in informal markets where fish is commonly sold and consumed. Informal roadside fish markets are, however, almost absent from this literature, and the extent to which post harvest handling adds to the contaminant load of the tissue that is actually eaten is unknown. This study assessed microplastic and element contamination in ready to eat braaied (open fire grilled) fish sold by roadside vendors in Thohoyandou, Limpopo Province, South Africa. Fish samples (*n* = 4 per stall) were collected from nine stalls, and microplastics extracted from the dorsolateral white muscle were characterised by morphology, colour and polymer type using stereomicroscopy and micro–Fourier transform infrared (µ–FTIR) spectroscopy, while element concentrations in the same tissue were determined by inductively coupled plasma mass spectrometry (ICP–MS) following microwave assisted acid digestion. The study indicates high microplastic burdens, dominated by fibres, films, and fragments. Total microplastic abundances differed significantly among sites, with mean values ranging from ~ 105 particles kg^− 1^ at site 9 to ~ 470 particles kg^− 1^ at site 1. Transparent microplastics dominated, and colours such as black, blue, red, yellow, green and other occurred at much lower concentrations. Polymer identification by µ–FTIR showed that polyethylene (40.5%), polypropylene (23.8%), polystyrene (18.3%) and polyvinyl chloride (7.4%) accounted for most of the particles analysed. Iron (Fe) was the dominant trace element, showing the highest mean concentrations across all sites (~ 70–110 mg kg^−1^). The concentrations of the 12 elements recorded in braaied fish from roadside vendors varied among sites, and mean Cr, Cu, Se and Mo concentrations exceeded FAO/WHO recommended maximum levels for fish at several stalls. Estimated consumer intake ranged from ~ 180 to ~ 790 particles per meal (~ 2–12 particles kg^− 1^ body weight), with the highest values at stalls adjacent to busy roads. There is a need for improved hygiene practices, better control of plastic pollution, and enhanced food safety monitoring in informal fish markets in South Africa.

**Supplementary Information:**

The online version contains supplementary material available at https://doi.org/10.1007/s00244-026-01223-3.

## Introduction

Plastic pollution is one of the defining environmental challenges of the Anthropocene, with global plastic production exceeding 400 million tonnes annually and projections suggesting continued growth in the coming decades (Geyer et al. 2017; Plastics Europe 2024). A major consequence of this production and subsequent waste mismanagement is the generation of microplastics, typically defined as plastic particles smaller than 5 mm, which are now widely recognised as ubiquitous in aquatic and terrestrial ecosystems (Dissanayake et al. 2022; O’Brien et al. 2023; Ali et al. 2024). Due to their small size and buoyancy, microplastics can be ingested or taken up via the gills by a broad range of organisms, including commercially important fish species, leading to ecological and food safety concerns (Barboza et al. 2018; Garrido–Gamarro et al. 2020; Dalu et al. 2024).

Fish are particularly vulnerable to microplastic ingestion through feeding and respiration, with several studies confirming the occurrence of microplastics in the gastrointestinal tract, gills, and, in some cases, edible tissues of marine and freshwater fish (Vidal et al. 2021; Wootton et al. 2021; Dalu et al. 2023, 2024; Sacco et al. 2024). These findings are significant because fish represent a major source of dietary protein for millions of people worldwide, and contamination by microplastics raises pressing questions about human exposure (Alberghini et al. 2022). Research has shown that microplastics can translocate from the gastrointestinal tract into edible tissues, potentially increasing direct exposure risk for consumers (Avio et al. 2015; Abbasi et al. 2018). Although most published studies focus on fish harvested through formal supply chains such as aquaculture, fisheries landing sites and supermarkets (e.g., Karami et al. 2017; Dalu et al. 2023, 2024; Sacco et al. 2024), there remains limited information about fish obtained from informal vendors, particularly roadside sellers, which are an essential source of protein in many low– and middle–income countries.

Roadside fish vending poses unique challenges for food safety assessment (Simiyu et al. 2021; Mushi 2023). In contrast to formal retail environments, where cold chain management and sanitary handling are often better regulated, roadside vendors frequently operate under conditions that may predispose fish to additional contamination (Lazaro et al. 2019). For example, fish displayed in open air near busy roads may be exposed to airborne microplastics derived from tyre wear, road dust, and/or nearby waste disposal activities (Wang et al. 2021; Rednikin et al. 2024). In addition, widespread use of plastic packaging materials, bags, and containers in informal markets introduces additional routes for microplastic contamination through surface contact or mechanical abrasion (Lau and Wong 2000; Sharma 2024). These pathways are seldom considered in studies that analyse fish contamination strictly within the aquatic environment (e.g., Naidoo et al. 2020; Wootton et al. 2021; Sacco et al. 2024), and the burden of microplastics in fish sold by informal roadside vendors may reflect both environmental accumulation in fish prior to harvest and post harvest contamination during handling, storage, and display. In Limpopo Province, street vending is widespread and culturally embedded, providing an important source of income for many households. Vendors sell food, beverages, clothing and household goods along roads and at transport nodes, and this vibrant informal economy also generates large quantities of single use plastic, including carrier bags, takeaway containers, disposable cutlery and polyethylene terephthalate (PET) bottles. Together with retail packaging waste, littering associated with high pedestrian traffic and limited waste management infrastructure in densely populated peri–urban settlements, this creates a plastic–rich environment in which food is prepared and sold (Crush and Frayne 2011; Premarathna et al. 2025).

Metal contamination in aquatic food systems is an escalating food safety concern, particularly within informal vending sectors where environmental exposure and processing conditions remain poorly regulated (Barboza et al. 2018). Fish are well known to bioaccumulate toxic metals such as mercury (Hg), cadmium (Cd), lead (Pb), and arsenic (As) through trophic transfer and direct uptake, often reaching concentrations that exceed permissible consumption thresholds (Saleem et al. 2022). Recent studies (e.g., Ali et al. 2019) further highlight that anthropogenic pressures, including industrial discharge, urban runoff, and atmospheric deposition, drive elevated metal burdens in fish tissues, with elevated concentrations exceeding regulatory limits in multiple regions. Importantly, emerging evidence indicates that processing methods commonly used in informal markets, such as open–fire grilling or traditional smoking, can introduce or amplify contamination, thereby increasing human exposure risks (Bello et al. 2026). While microplastics may facilitate metal transport, current research suggests that direct environmental contamination and post harvest handling pathways remain the dominant determinants of metal loads in fish (Wang et al. 2024). In this context, the present study quantifies element concentrations alongside microplastic characteristics in braaied fish from roadside vendors, and evaluates how the environmental context of the vending site relates to contamination profiles and associated exposure.

Informal food systems play a central role in daily nutrition for urban and peri–urban populations, yet they remain understudied with respect to contaminant exposure risks (Grace 2015). Studying microplastic contamination in fish sold by informal vendors is important as informal food systems are critical to food and nutrition security in many regions of the Global South, where fish is an affordable source of animal protein that is often purchased from roadside vendors rather than supermarkets and/or formal wet markets (Crush and Frayne 2011). Furthermore, the influence of cooking practices is highly relevant to exposure assessment, as heat treatment can degrade or modify microplastics, with implications for bioavailability in humans (Morgana et al. 2018). Consumers in these contexts may therefore be exposed to microplastics repeatedly, at levels that have not yet been quantified. Furthermore, while most studies (e.g., Karami et al. 2017; Abbasi et al. 2018) report microplastics in non–edible organs such as the gastrointestinal tract, which are commonly discarded before consumption, few studies have examined edible tissues within informal food systems (but see Simiyu et al. 2021; Mushi 2023). Establishing the extent of contamination in fish muscle tissue or skin, which are typically consumed, is essential for a more accurate assessment of dietary exposure. Thus, the informal nature of roadside vending introduces additional risk factors that are not adequately captured in existing research, as vendors often operate without refrigeration, protective display infrastructure, or standardised packaging, all of which may increase the risk of contamination (Simiyu et al. 2021; Mushi 2023). Environmental factors, such as proximity to industrial zones, traffic corridors, and waste disposal sites, further increase the likelihood of microplastic contamination in fish products. By analysing contamination in this specific context, the combined contribution of aquatic exposure and post harvest contamination can be evaluated, providing a more complete understanding of the contamination pathway.

This work carries clear policy relevance as current debates on microplastics in food systems remain largely focused on marine capture fisheries, aquaculture, and bottled or packaged products (Smith et al. 2018). Yet, in many countries, informal vendors supply a substantial share of daily food intake, often for the most economically vulnerable populations. Generating evidence on contamination levels in these settings could inform targeted risk communication, regulatory interventions on plastic use in informal markets, and consumer protection policies. Moreover, such data could support broader waste management and pollution mitigation strategies, aligning with Sustainable Development Goal 12 on sustainable consumption and production.

The present study aims to assess the extent and characteristics of microplastic and element contamination in fish consumed from informal roadside vendors by quantifying microplastic abundances and element concentrations in braaied fish muscle, characterising the particles (i.e., morphology, colour and polymer composition), and evaluating variation in contamination in relation to the environmental context of the vending stall. By linking observed contamination to potential dietary exposure, the study provides a preliminary, screening level estimate of dietary exposure for consumers who depend on informal fish markets. It is hypothesized that (i) fish sold by informal roadside vendors will exhibit significant contamination by microplastics and elements in edible tissues, and (ii) microplastic and element contamination levels will vary significantly according to the environmental context of the stall, with higher concentrations in fish sold near busy roads and poorly managed waste sites.

## Materials and Methods

### Study Area

**Fig. 1:**
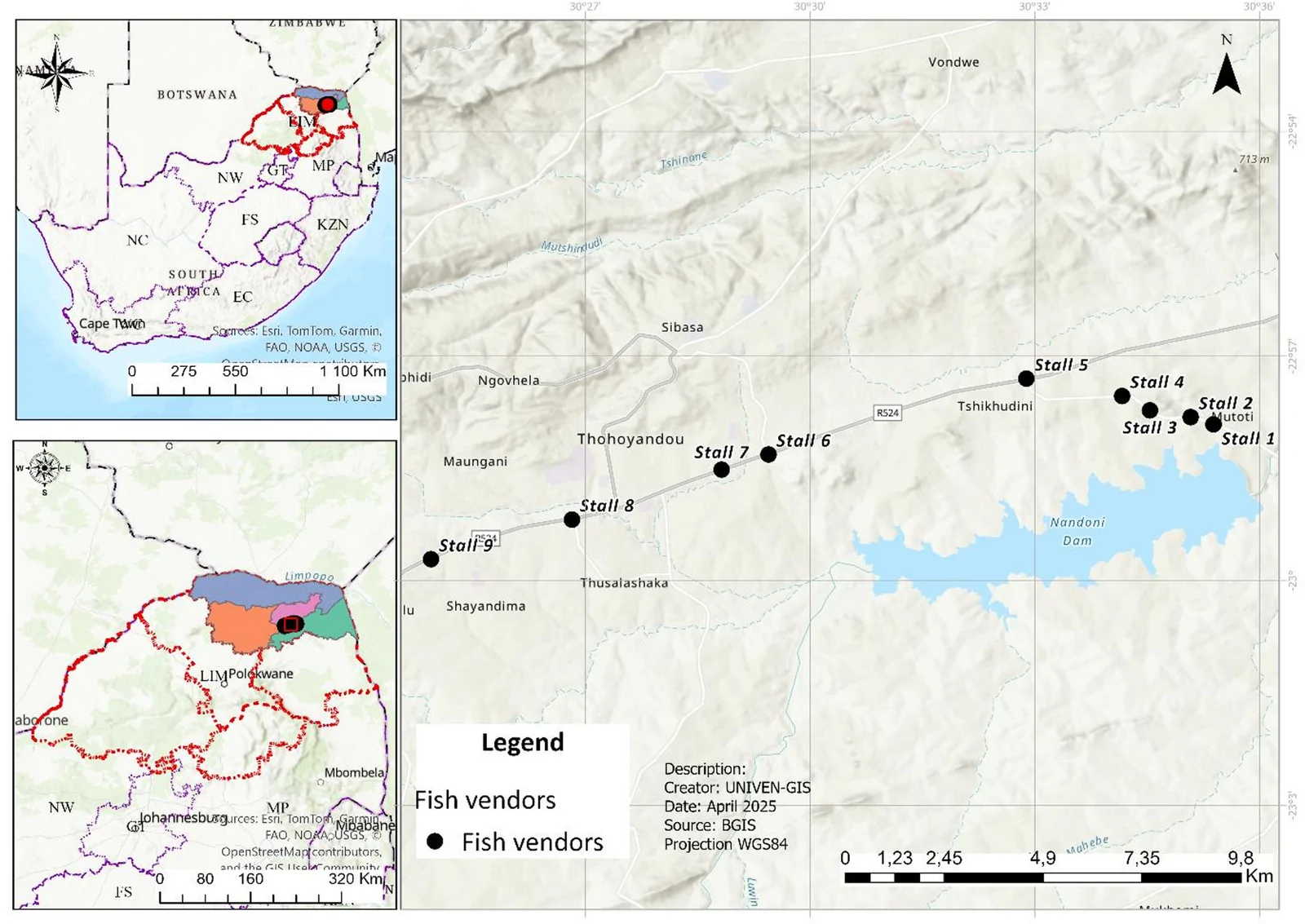
Location of the nine roadside fish–vending stalls sampled in relation to Nandoni reservoir, Thohoyandou, South Africa

This study was conducted in Thohoyandou, the administrative and commercial centre of the Thulamela Local Municipality and Vhembe District, Limpopo Province, South Africa (Fig. 1). Thohoyandou is situated at an elevation ranging between 600 and 700 m above sea level. The town lies in a subtropical climatic region, characterised by hot, humid summers (October–March) with mean annual rainfall of 800–1200 mm, and cool–dry winters (May–August). Extensive areas of the surrounding landscape have been converted to agricultural land, settlements and commercial hubs, and the informal vending sector described above is concentrated along the main road network. Stormwater runoff during the rainy season further facilitates the transport of mismanaged plastic waste into nearby streams and drainage systems, raising concerns about downstream impacts on aquatic and terrestrial ecosystems. The nine vending stalls sampled were distributed along the R524 and adjoining roads between Thohoyandou and Nandoni Dam (Fig. 1). Stalls 1–5 were situated on the busier eastern section of the R524 closer to Nandoni Dam, whereas stalls 6–9 were located closer to the Thohoyandou central business district. Typical vending conditions are illustrated in Fig. S1.

### Sampling

Fish samples (*n* = 4 per stall; 36 fish in total) were bought from nine roadside vending stalls along the R524 and adjoining roads within the Thulamela Municipality region during August 2025 (Fig. 1). All stalls trading along this road network on the sampling dates were included, so the nine stalls represent a complete census of the accessible roadside fish vendors rather than a random subsample; their linear arrangement in Fig. 1 therefore reflects the layout of the road itself. At each stall, four fish were selected at random from those displayed for sale. All fish were purchased ready to eat, having been braaied (the southern African term for grilling over an open wood or charcoal fire) by the vendor and displayed uncovered on open grills or trays. The fish sampled were mostly *Oreochromis niloticus* × *Oreochromis mossambicus* hybrids. Each fish was individually packaged in a labelled paper bag, transported to the laboratory within 48 h of purchase and stored at freezer until processing.

The standard length (SL, cm) for all the fish samples was measured from the tip of the snout to the longest caudal fin ray. The dissection to obtain muscle tissue was conducted with stainless steel instruments rinsed in ultrapure MilliQ distilled water and rinsed with ethanol between samples. To exclude element contamination from the dissection tools, all stainless steel instruments were acid washed (10% v/v HNO_3_, 24 h) and rinsed with 18 MΩ·cm ultrapure water before use. All samples were covered and the air conditioning was switched off to prevent contamination. In all fish specimens, the collected defined muscle portion was the dorsolateral white muscle on the left side between the operculum and the dorsal fin to ensure consistency. Each fish was then divided into two sub–samples. The sub–sample designated for element analysis was oven–dried at 60 °C for 72 h to constant mass and homogenised to a fine powder using an acid–washed agate mortar and pestle (Saleem et al. 2022). The subsample designated for microplastic analysis was retained wet, weighed and processed immediately by alkaline digestion, because drying and grinding can fragment particles and inflate counts (Karami et al. 2017; Dalu et al. 2023).

### Pre–Analysis Handling and Contamination Minimisation

To reduce external contamination of microplastic samples, all sample handling and processing were conducted in a controlled laboratory environment. Work surfaces were cleaned with filtered ultrapure MilliQ deionised water and covered when not in use. Personnel wore natural fibre laboratory coats and, where possible, non–synthetic laboratory coats. Powder–free nitrile gloves were worn at all times; nitrile does not contribute the polymer types quantified here (i.e., polyethylene, polypropylene, polystyrene and polyvinyl chloride), and any glove–derived particles would have been captured by the procedural blanks. All solutions and rinse water were filtered through 0.2 μm filters prior to use. Procedural field and laboratory blanks (*n* = 5) were processed alongside every batch of samples to quantify airborne and laboratory contamination. Blanks contained a mean of 2 ± 1 particles per blank, consisting mainly of blue fibres. The mean blank count was subtracted from the corresponding sample counts, the limit of detection was defined as the mean blank count plus three standard deviations, and particle categories that did not exceed this threshold in samples were not reported. Where practical, filtration and visual sorting were performed under a laminar flow cabinet, and all glassware was rinsed with filtered solvent and covered between uses. Sample containers and tools were made of glass or metal, with the choice of materials and any potential contamination effects documented. For the element analysis, all glass and polytetrafluoroethylene ware was soaked in 10% (v/v) HNO₃ for 24 h and rinsed three times with 18 MΩ·cm ultrapure water before use, reagent blanks were digested and analysed with each batch of samples, and only trace–metal–grade reagents were used.

### Sample Preparation for Microplastic Analysis

Fish muscle tissues (weight range = 65.2–102.6 g per fish) cut for microplastic analysis underwent organic matrix removal and particle separation using established density and chemical digestion approaches appropriate for preserving synthetic polymers (Avio et al. 2015; Karami et al. 2017; Dalu et al. 2023). The laboratory method relied on enzymatic or alkaline digestion to remove proteinaceous material while minimising polymer degradation. The digestion parameters were selected based on literature evidence for effective tissue removal and polymer preservation (Erdőssy et al. 2017). In brief, the microplastic extraction process began by transferring a measured portion of fish muscle into a clean digestion vessel, where it was treated with 10% (w/v) potassium hydroxide (KOH; 98% analytical grade, Chemicorp, Barbeton) at a ratio of about 10:1 (v/w). The mixture was incubated at 40–60 °C for 24–48 h with gentle agitation until the tissue is fully broken down. Following digestion, a high–density solution (zinc chloride, ZnCl_2_, density 1.6–1.7 g cm^− 3^; Chemicorp, Barbeton) was used for density separation to concentrate buoyant and near neutral density particles. Suspensions were then passed through mixed cellulose ester filters (diameter 42 mm, pore size 0.45 μm; Filter-Bio, Nantong City) and filters were retained for particle enumeration and characterisation based on type (i.e., beads, fibres, fragments, films) and colour (i.e., black, blue, red, yellow, green, transparent and other) using an Olympus stereomicroscopy at 50–200× magnification as part of an initial triage for identification. Particle abundance was expressed relative to tissue wet mass and is reported throughout as particles kg^− 1^ wet mass. Particle size classes were not quantified in the present study (see Limitations).

Polymer identification of assumed microplastics was conducted using spectroscopic methods to reduce misclassification. Selected particles (i.e., representative of morphological and colour classes, and all suspected particles above a size threshold) were analysed by micro–Fourier transform infrared spectroscopy (µ–FTIR) in micro–transmission mode to assign polymer types. The equipment used was a PerkinElmer Spotlight 200 Spectrum Two FTIR microscope fitted with a mercury cadmium telluride (MCT) detector (PerkinElmer Inc., Waltham, MA, USA) and a Thermo Scientific Nicolet iS20 FTIR spectrometer (Thermo Fisher Scientific, Madison, WI, USA). To do this, the particles analysed were placed on a potassium bromide (KBr) disc, which was used as a slide, and their spectra were recorded in micro-transmission mode using the following parameters: spot 50 μm, 32 sweeps and a spectral range of 550–4 000 cm^− 1^. The spectra obtained were analysed using OMNIC software (Thermo Scientific; version 9.13.1256) and data libraries. We measured the spectra for each particle, compared them to a validated spectral library, and reported matches with quality indices, such as the polymer library match score > 70–80%, as acceptable. In total, 60% of particles counted visually were analysed by µ–FTIR, of which 98% were confirmed as synthetic polymers. Visually identified particles that were not confirmed as synthetic polymers were excluded from the reported abundances, and the confirmation rate was applied to the remaining unanalysed particles.

### Sample Preparation for Element Analysis

Target elements included sodium (Na), magnesium (Mg), phosphorus (P), potassium (K), calcium (Ca), chromium (Cr), manganese (Mn), iron (Fe), copper (Cu), zinc (Zn), selenium (Se) and molybdenum (Mo) were selected because they are readily released from vehicle exhaust emissions and road dust and could therefore affect roadside sold fish (Onjefu et al. 2017). We used acid digestion followed by inductively coupled plasma mass spectrometry (ICP–MS) at the Central Analytical Facility at Stellenbosch University. Using a separate aliquot of muscle of approximately 10 g dry weight, the tissues were placed in a polytetrafluoroethylene digestion vessel for microwave–assisted digestion, combined with a digestion mixture of 6–8 mL concentrated ultrapure nitric acid (HNO_3_, trace metal grade) and 1–2 mL hydrogen peroxide (30%) to assist oxidation. These were digested using a program ramping to 180–200 °C for 15–30 min, with a hold following the instrument/acid vendor and safety guidance. After cooling, we quantitatively transferred the digestate to acid cleaned polypropylene tubes and diluted to a final volume of 50 mL with 18 MΩ·cm ultrapure MilliQ water. The diluted digests were analysed for multi element determination on an ICP–MS at the Stellenbosch University Central Analytical Facility. Calibration was performed with Ref Fish 39 Tort-3 certified multi element standard, and all calibration curves had *r*^2^ > 0.999. The certified reference material TORT–3 (fish 39, National Research Council Canada), reagent blanks and duplicate samples were digested and analysed with every batch. The machine %accuracy and %recovery ranged from 93.3 to 108.7% and 95.3–105.4%, respectively. Limits of detection and quantification were calculated as three and ten times the standard deviation of the reagent blanks, respectively, and all reported concentrations exceeded the limit of quantification. Concentrations are reported as mg kg^− 1^.

### Data Analysis

#### Estimation of Microplastic Intake from Braaied Fish

Microplastic intake from fish consumption was estimated using experimentally measured microplastic concentrations in the edible muscle tissue. First, the number of particles ingested per meal was calculated by multiplying the mean microplastic concentration of each fish sample (expressed as particles g^− 1^ wet weight) by a representative portion size, expressed in grams of edible tissue. This yielded a standardized intake metric for a single eating event: The approach follows that used in previous seafood exposure assessments (Smith et al. 2018; Al Mamun et al. 2023).

Intake per meal (particles) = mean microplastic concentration (particles g^− 1^ edible tissue) × typical portion size (g).

Daily fish consumption values were derived from South African per capita estimates reported for 2021, corresponding to 6.6 kg·capita^− 1^ year^− 1^. This value was converted to a daily intake rate by dividing by 365, yielding an estimated 18.08 g of fish per person per day. This value was used to contextualize the per meal intake relative to typical national consumption patterns.

To standardise exposure across individuals of different body masses, microplastic intake was further normalized to body weight. An average consumer body weight of 71 kg for adults was assumed. Because body mass varies widely between children and adults, and roadside fish are also eaten by transient consumers, intake is additionally expressed per kilogram of body weight so that exposure can be rescaled for any consumer group. Intake per kilogram of body weight was calculated by dividing the number of particles ingested per meal by the consumer’s body weight (kg):

$$\:\mathrm{Microplastic\:intake\:}\left({\mathrm{particles\:kg}}_{bw}^{-1}\right)=\frac{{\mathrm{Intake}}_{\mathrm{per\:meal}}}{\mathrm{body\:weight\:(kg)}}$$

Where, bw – body weight. This approach provides both an absolute estimate of microplastic exposure per consumption event and a body–weight–adjusted metric suitable for comparative exposure assessment. No toxicological reference dose currently exists for microplastic particles, so these values are presented as screening level exposure estimates rather than as a formal health risk characterisation (Smith et al. 2018; Leslie and Depledge 2020). For the same reason, element concentrations were compared with FAO/WHO maximum permissible levels for fish rather than being converted into estimated daily intakes, target hazard quotients and hazard indices, because oral reference doses are not available for all of the elements measured here.

#### Statistical Analysis

All statistical analyses were conducted to evaluate spatial variation in contaminant loads and to explore biometric predictors of microplastic and element concentrations in braaied fish muscle. Prior to analysis, all datasets were examined for normality using Shapiro–Wilk tests and Q–Q plots, and for homogeneity of variances using Levene’s test. The data were found to meet all assumptions, and a one–way analysis of variance (ANOVA) was used to test whether concentrations of individual microplastic types (e.g., fibres, fragments, films, beads) and elements differed significantly among the nine sampling sites (i.e., sites 1–9). Sites were treated as a fixed categorical factor. For each response variable, ANOVA models were fitted independently, and significant omnibus effects (*p* < 0.05) were followed by Tukey’s HSD post–hoc comparisons to identify specific site pairs that differed. With four fish per stall (*n* = 36), the design has limited power to detect small differences among stalls, so only comparatively large effects would have been detected and non–significant results should not be interpreted as evidence of no difference.

To assess whether fish size influenced contaminant loads, linear regression models were developed using standard length (cm) and total weight (g) as continuous predictors. Separate regression models were fitted for each response variable, including abundances of microplastic categories and concentrations of individual elements measured in muscle tissue. Multicollinearity between length and weight was evaluated using variance inflation factors (VIFs), and predictors were retained only when VIF < 5 to avoid parameter estimation bias. Model diagnostics, including residual plots, Cook’s distance, and tests for heteroscedasticity, were used to verify regression assumptions. Where appropriate, generalised linear models (GLMs) with Poisson or negative binomial error structures were applied to microplastic count data to account for overdispersion or non–normal distributions. For element concentrations exhibiting skewed distributions, log transformed values were used in the regression models. All statistical results were reported with corresponding *p*–values, coefficients (β), confidence intervals, and measures of model fit (R^2^ or pseudo–R^2^ as appropriate). Statistical analyses were performed in SPSS version 25 (IBM Corp., Armonk, NY, USA), and the significance level was set at *p* < 0.05.

## Results

### Microplastics

**Fig. 2:**
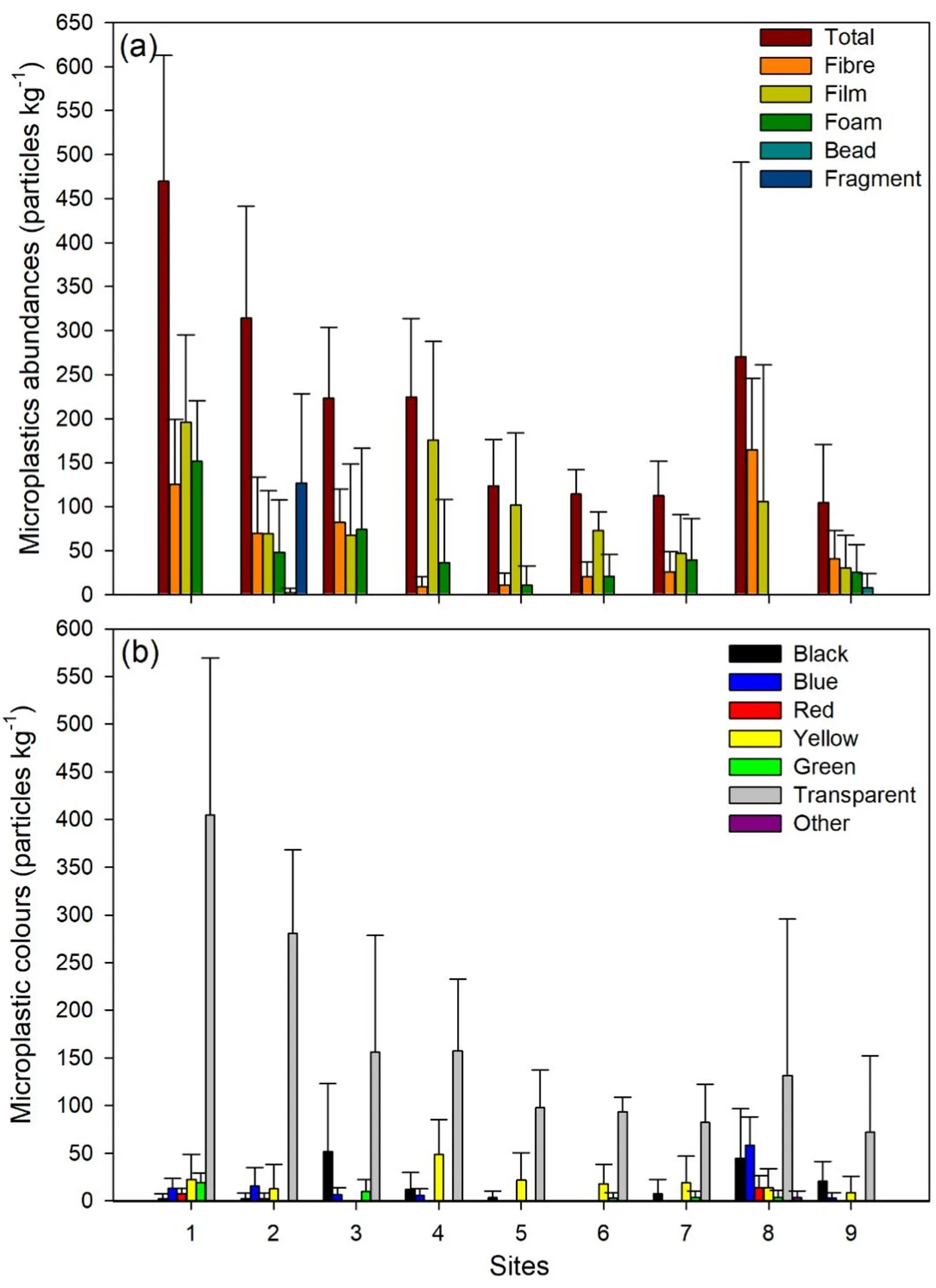
Microplastic **a** morphotypes and **b** colours in the muscle of braaied fish among the different selling stalls in the Thulamela Municipality region. Bars show mean values (*n* = 4 fish per stall) and error bars represent one standard deviation

Across all nine sampling sites, microplastics were consistently present in the edible muscle of braaied fish, although their abundances varied widely among sites (Fig. 2). The highest mean total microplastic abundance was recorded at site 1 (~ 470 particles kg^− 1^), followed by sites 2 (~ 315 particles kg^− 1^) and 8 (~ 270 particles kg^− 1^), while the lowest mean abundances were recorded at sites 6, 7 and 9 (~ 105–110 particles kg^− 1^) (Fig. 2a). Fibres and films were the dominant morphotypes overall, although their relative proportions differed among stalls: mean film abundances were highest at sites 1, 4 and 8, and mean fibre abundances at sites 1, 3 and 8 (Fig. 2a). Fragments were recorded almost exclusively at site 2 (~ 127 particles kg^− 1^), and beads occurred only at low mean abundances at sites 2 and 9 (Fig. 2a). Based on the one–way ANOVA analysis, total microplastic abundances differed significantly among sites (F = 4.905, *p* = 0.001), indicating that overall microplastic loads varied considerably. Among the morphotypes, fibres, foam, and fragments all showed significant site related variation (*p* < 0.05). Fibres (F = 5.412, *p* < 0.001) and fragments (F = 6.264, *p* < 0.001) exhibited the strongest site effects, while foam showed moderate but statistically meaningful variation (F = 2.817, *p* = 0.021). In contrast, films (*p* = 0.136) and beads (*p* = 0.509) did not differ significantly across sites, indicating no detectable difference among stalls for these particle types (Table 1).

In terms of colour distribution, transparent microplastics dominated across all sites (Fig. 2b). Site 1 showed the highest mean abundance, with transparent particles exceeding 400 particles kg^− 1^, followed by sites 2 and 3, where levels ranged from 200 to 300 particles kg^− 1^. Black, blue, red, yellow, green and other particles occurred at much lower mean concentrations, generally below 50 particles kg^− 1^ (Fig. 2b). Black and blue particles were more common than the other colour categories, particularly at sites 2 and 8, but they remained a small fraction compared to transparent particles. For microplastic colours, several categories also varied significantly among sites (Table 1). Blue particles (F = 8.123, *p* < 0.001), red particles (F = 4.411, *p* = 0.002), green particles (F = 3.464, *p* = 0.007), and transparent particles (F = 4.749, *p* = 0.001) all showed significant spatial differences, reflecting strong local variation in microplastic sources or exposure pathways. In contrast, black, yellow, and other colours did not differ significantly among sites (*p* > 0.05) (Table 1).

From the µ–FTIR results for polymer identification, polyethylene (40.5%), polypropylene (23.8%), polystyrene (18.3%) and polyvinyl chloride (7.4%) dominated most of the plastic polymers identified in the study.

**Table 1:** One–way analysis of variance for fish microplastic morphotypes, colours and elements (df = 8 for all tests; *n* = 36 fish). Asterisks indicate significance at *p* < 0.05. Values of *p* below 0.001 are reported as < 0.001, as returned by the software

Microplastics	df	F	*p*	Elements	df	F	*p*
Total	8	4.905	0.001*	Na	8	9.587	< 0.001*
*Morphotypes*				Mg	8	0.941	0.500
Fibres	8	5.412	< 0.001*	P	8	0.711	0.679
Film	8	1.734	0.136	K	8	0.734	0.661
Foam	8	2.817	0.021*	Ca	8	0.819	0.593
Bead	8	0.929	0.509	Cr	8	6.402	< 0.001*
Fragment	8	6.264	< 0.001*	Mn	8	13.961	< 0.001*
*colours*				Fe	8	20.371	< 0.001*
Black	8	1.498	0.204	Cu	8	1.246	0.311
Blue	8	8.123	< 0.001*	Zn	8	0.946	0.497
Red	8	4.411	0.002*	Se	8	5.501	< 0.001*
Yellow	8	1.200	0.336	Mo	8	10.351	< 0.001*
Green	8	3.464	0.007*				
Transparent	8	4.749	0.001*				
Other	8	1.000	0.459				

**Fig. 3:**
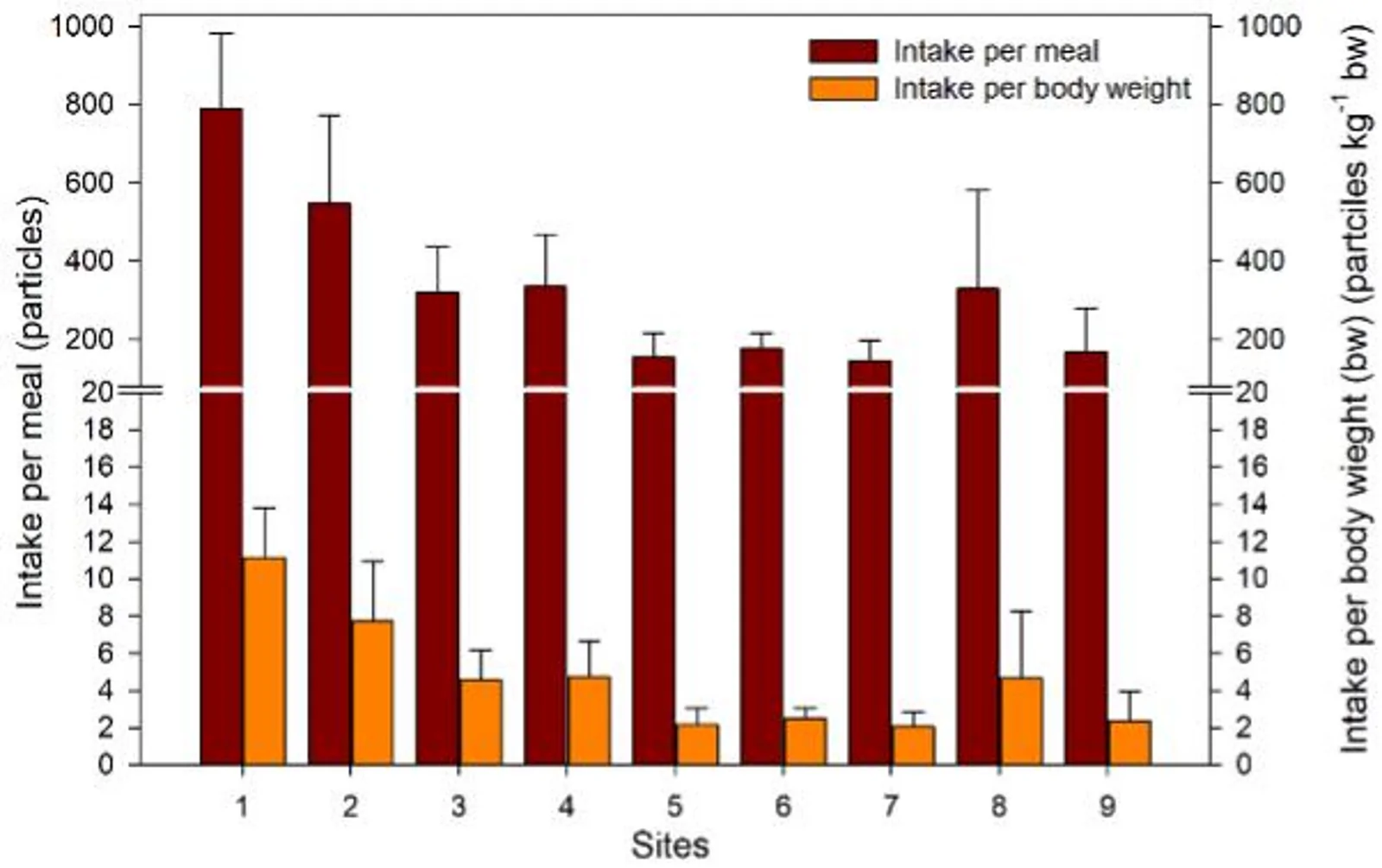
Variation in the estimated microplastic intake rates per meal and per kilogram of body weight among the different study sites. Bars show mean values (*n* = 4 fish per stall) and error bars represent one standard deviation

Estimated microplastic intake differed among stalls (Fig. 3). The highest mean intake was estimated for site 1 (~ 790 particles per meal; ~11 particles kg^−1^ bw), followed by site 2 (~ 550 particles per meal; ~8 particles kg^−1^ bw). Sites 3, 4 and 8 gave intermediate mean values (~ 330–350 particles per meal; ~4–6 particles kg^−1^ bw), and sites 5–7 and 9 the lowest (< 200 particles per meal; < 3 particles kg^−1^ bw). Normalising to body weight preserved the same ranking among stalls.

For standard length, all morphotypes except beads showed significant negative relationships with fish size (Table 2). High counts of fibres, films, foams and fragments were associated with small fish. Beads also exhibited a negative trend, but the relationship was marginal (*p* = 0.060). All morphotypes, including beads, were positively and significantly associated with body mass (Table 2). Large fish therefore contained greater numbers of microplastic particles overall, particularly fibres, films, foams and fragments (Table 2).

Microplastic colours showed a similar but directionally opposite pattern to morphotypes (Table 2). For standard length, most colours such as black, blue, yellow, green, other and transparent colour were positively related to fish length, which indicated that large fish contained more particles of these colours. Red particles showed no significant (*p* > 0.05) relationship (Table 2). For weight, the model showed an opposing trend, with nearly all colours, with the exception of red, had a significant negative association with body mass (Table 2). Thus, larger fish carried fewer coloured particles per unit body mass than smaller individuals (Table 2).

**Table 2:** Linear regression of fish standard length and body weight against microplastic morphotype and colour abundances and element concentrations. *β* ± SE are unstandardised coefficients and β the standardised coefficient. Asterisks indicate significance at *p* < 0.05

Variable	Standard length					Weight			
	β ± SE	β	t	*p*	β ± SE		β	t	*p*
Constant	21.54 ± 6.64		3.240	0.008*	97.98 ± 24.78			3.954	0.002*
*Microplastic morphotypes*									
Fibre	− 0.17 ± 0.04	− 4.509	− 3.539	0.005*	0.63 ± 0.18		4.064	3.510	0.005*
Film	− 0.14 ± 0.04	− 5.293	− 3.104	0.010*	0.5 ± 0.17		4.452	2.873	0.015*
Foam	− 0.13 ± 0.04	− 3.430	− 3.045	0.011*	0.48 ± 0.16		2.996	2.926	0.014*
Bead	− 0.18 ± 0.08	− 0.406	− 2.097	0.060	0.87 ± 0.32		0.471	2.677	0.022*
Fragment	− 0.14 ± 0.04	− 2.855	− 3.027	0.012*	0.52 ± 0.17		2.530	2.953	0.013*
*Microplastic colours*									
Black	0.16 ± 0.04	2.139	3.331	0.007*	− 0.57 ± 0.18		− 1.839	− 3.152	0.009*
Blue	0.23 ± 0.06	1.962	3.619	0.004*	− 0.92 ± 0.24		− 1.862	− 3.779	0.003*
Red	0.13 ± 0.14	0.338	0.958	0.359	− 0.49 ± 0.52		− 0.303	− 0.947	0.364
Yellow	0.12 ± 0.04	1.241	2.962	0.013*	− 0.41 ± 0.15		− 1.001	− 2.629	0.023*
Green	0.25 ± 0.07	0.829	3.241	0.008*	− 0.91 ± 0.28		− 0.741	− 3.188	0.009*
Transparent	0.15 ± 0.04	8.276	3.272	0.007*	− 0.53 ± 0.17		− 7.091	− 3.085	0.010*
Other	0.77 ± 0.22	0.705	3.440	0.006*	− 3.1 ± 0.83		− 0.693	− 3.720	0.003*
*Elements*									
Na	0 ± 0	− 0.342	− 2.011	0.069	0 ± 0		0.298	1.925	0.081
Mg	− 0.012 ± 0	− 1.003	− 2.361	0.038*	0.045 ± 0.01		0.910	2.358	0.038*
P	0.001 ± 0	2.064	2.114	0.058	− 0.0028 ± 0		− 2.399	− 2.704	0.020*
K	− 0.001 ± 0	− 0.299	− 1.578	0.143	0.0012 ± 0		0.180	1.044	0.319
Ca	0 ± 0	− 0.340	− 0.521	0.613	0.0003 ± 0		0.557	0.939	0.368
Cr	− 17.01 ± 2.77	–1.595	− 6.131	< 0.001*	50.42 ± 10.34		1.152	4.876	< 0.001*
Mn	0.01 ± 0.59	0.015	0.033	0.974	− 0.14 ± 2.23		− 0.027	− 0.065	0.949
Fe	− 0.1 ± 0.02	–1.111	− 4.014	0.002*	0.38 ± 0.09		1.013	4.030	0.002*
Cu	2.4 ± 1.53	0.274	1.567	0.145	− 18.55 ± 5.72		− 0.516	− 3.240	0.008*
Zn	− 0.1 ± 0.07	–0.438	− 1.402	0.189	0.43 ± 0.26		0.462	1.626	0.132
Se	6.08 ± 2.2	1.000	2.765	0.018*	− 29.58 ± 8.2		− 1.186	− 3.607	0.004*
Mo	342.28 ± 64.92	1.793	5.272	< 0.001*	− 823.28 ± 241.93		− 1.051	− 3.403	0.006*

### Elements

**Fig. 4:**
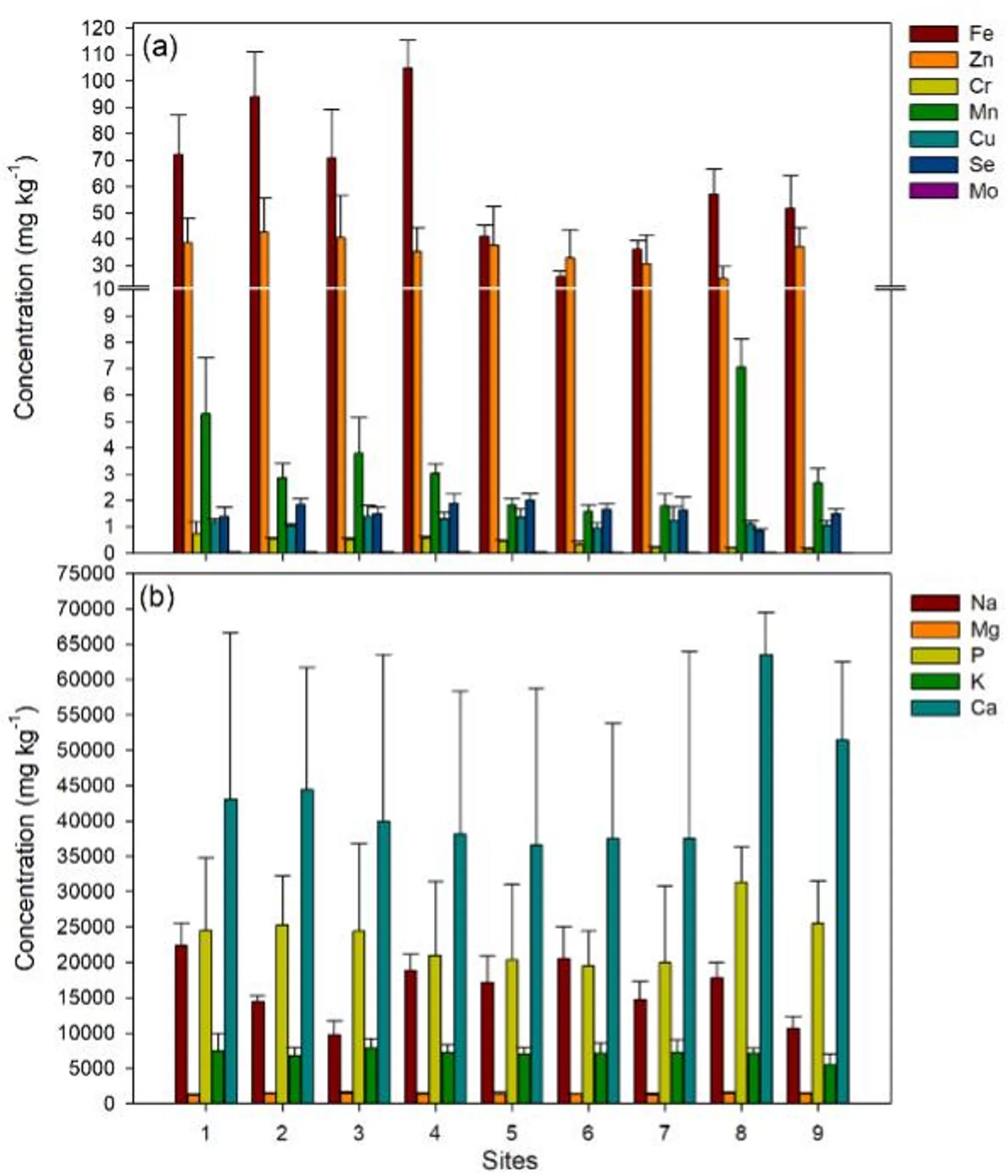
Element concentrations in the muscle of braaied fish recorded at the various fish selling stalls: **a** trace elements and **b** macro–elements. Bars show mean values (*n* = 4 fish per stall) and error bars represent one standard deviation

Iron was the most abundant trace element at every stall (mean ~ 30–105 mg kg^− 1^), with the highest mean concentrations at sites 2 and 4 and the lowest at sites 6 and 7 (Fig. 4a). Zinc was the second most abundant trace element (mean ~ 20–45 mg kg^− 1^) and was comparatively uniform among stalls (Fig. 4a; Table 1). Mean manganese concentrations were generally < 4 mg kg^− 1^ but were markedly higher at sites 8 (~ 7 mg kg^− 1^) and 1 (~ 5 mg kg^− 1^). Mean chromium concentrations were low (< 1.5 mg kg^− 1^), with the highest mean value at site 1. Mean copper and selenium concentrations were also low (typically < 2 mg kg^− 1^) and varied little among stalls, and mean molybdenum concentrations were the lowest of the elements measured (< 1 mg kg^− 1^), although Mo nonetheless differed significantly among stalls (Fig. 4a; Table 1).

Macro–elements occurred at much higher concentrations than the trace elements, consistent with their physiological role in fish tissue (Fig. 4b). Calcium was the most abundant element (mean ~ 37 000–64 000 mg kg^− 1^), with the highest mean concentrations at sites 8 and 9 and high within–stall variability at nearly all stalls (Fig. 4b). Mean phosphorus concentrations ranged from ~ 19 000 to ~ 31 000 mg kg^− 1^ and were highest at site 8. Mean sodium concentrations ranged from ~ 10 000 to ~ 22 000 mg kg^− 1^, with the highest means at sites 1 and 6 and the lowest at sites 3 and 9. Mean potassium concentrations were comparatively uniform among stalls (~ 7 000–8 000 mg kg^− 1^), as were mean magnesium concentrations, which were the lowest of the macro–elements (~ 1 000–1 600 mg kg^− 1^) (Fig. 4b).

Based on the one–way ANOVA, element concentrations displayed more pronounced spatial heterogeneity (Fig. 4; Table 1). Several elements, including Na, Cr, Mn, Fe, Se, and Mo, showed highly significant effects (*p* < 0.001). These elements therefore differed substantially among the stalls sampled. In contrast, Mg, P, K, Ca, Cu, and Zn did not vary significantly among sites (*p* > 0.05), indicating no detectable difference among stalls for these elements (Table 1).

From the regression analysis, element–size relationships were more variable (Table 2). Magnesium showed a weak negative and significant (*p* < 0.05) relationship, while Cr, Fe and Mo exhibited strong negative and significant (*p* < 0.05) associations, indicating that small fish had higher concentrations of these elements (Table 2). Selenium was positively and significantly associated with standard length, suggesting that larger fish accumulated more Se. For fish weight, several elements showed strong and significant (*p* < 0.05) effects, but in directions opposite to length (Table 2). Chromium and Fe showed strong positive and significant relationships, indicating that large fish contained more of these elements (Table 2). In contrast, Cu and Se were significantly and negatively associated with weight (*p* < 0.05), with higher concentrations in small fish. Molybdenum also showed a significant negative relationship (*p* < 0.05) with substantially lower levels in big fish (Table 2). Mean concentrations of Cr, Cu, Se and Mo exceeded the maximum levels recommended for fish at several stalls (Cr 1 mg kg^− 1^; Cu 30 mg kg^− 1^; Se 2 mg kg^− 1^; Mo 1 mg kg^− 1^). Iron is not assigned a maximum permissible level in fish and is reported for comparison only.

No statutory maximum level in fish has been established for any of the 12 elements measured, either by the Codex Alimentarius (CXS 193-1995) or under South African regulation (R.588 of 2018); measured concentrations were therefore compared with the screening values conventionally applied to fish (FAO 1983) and with available health‑based guidance values (Table 3). Mean copper (1.0–2.0 mg kg^− 1^), selenium (1.0–2.0 mg kg^− 1^) and molybdenum (< 1.0 mg kg^− 1^) concentrations were below their screening values at all nine stalls. Mean zinc concentrations exceeded the 40 mg kg^−1^ screening value at site 2 only. Mean chromium reached the 1 mg kg^− 1^ screening value at site 1 and remained between 0.3 and 0.5 mg kg^− 1^ elsewhere, and mean iron approached the 100 mg kg^− 1^ value at site 4 (~ 105 mg kg^−1^), remaining below it at the other eight stalls. Mean manganese concentrations exceeded the 0.5–1.0 mg kg^− 1^ screening value at all nine stalls and reached ~ 7 mg kg^− 1^ at site 8 (Table 3). The macro‑elements have no screening or statutory value in fish (Table 3). Mean sodium (10 000–22 000 mg kg^− 1^), calcium (37 000–64 000 mg kg^− 1^), phosphorus (19 000–31 000 mg kg^− 1^), potassium (7 000–8 000 mg kg^− 1^) and magnesium (1 000–1 600 mg kg^− 1^) concentrations were one to two orders of magnitude above published ranges for uncontaminated freshwater fish muscle. A 100 g portion of fish from the stalls sampled would deliver 1.0–2.2 g of sodium.

**Table 3:** Statutory maximum levels, health–based guidance values and screening values reported for the elements measured in this study. RNI, recommended nutrient intake; UL, tolerable upper intake level; PMTDI, provisional maximum tolerable daily intake; RfD, oral reference dose; AI, adequate intake. No statutory maximum level in fish exists for any of the elements measured or for microplastics under Codex CXS 193–1995, which sets maximum levels in fish only for Pb, Cd, inorganic As and methylmercury, or under the South African Foodstuffs, Cosmetics and Disinfectants Act (Act 54 of 1972), Regulation R.588 of 2018, which repealed the earlier Cu and Zn limits of R.500 of 2004. The values in the final column are those most often applied in the fish–contaminant literature and derive from FAO Fishery Circular No. 464 (1983), a compilation of national limits rather than an international standard; they are used here as screening benchmarks only

Element	Codex/FAO–WHO maximum level in fish	JECFA / WHO health–based guidance value	US EPA oral RfD (mg kg^− 1^ bw d^− 1^)	South Africa (*R*.588 of 2018)	Screening value commonly applied to fish (source)
Na	None	WHO: < 2 000 mg Na day^− 1^ (adults)	None	None	
Mg	None	FAO/WHO RNI ~ 220–260 mg day^− 1^	None	None	
P	None	No FAO/WHO value (IOM RDA 700 mg day^− 1^)	None	None	
K	None	WHO: ≥3 510 mg day^− 1^ (adults)	None	None	
Ca	None	FAO/WHO RNI ~ 1 000 mg day^− 1^ (adults)	None	None	
Cr	None	No JECFA value; FAO/WHO RNI 25–35 µg Cr(III) day^− 1^	1.5 (Cr III); 0.003 (Cr VI)	None	1.0 mg kg^− 1^ (FAO 1983)
Mn	None	No JECFA value	0.14	None	0.5–1.0 mg kg^− 1^ (FAO 1983)
Fe	None	JECFA PMTDI 0.8 mg kg^− 1^ bw d^− 1^	0.7	None	100 mg kg^− 1^ (FAO 1983)
Cu	None	JECFA PMTDI 0.05–0.5 mg kg^− 1^ bw d^− 1^	0.04	None (repealed in 2018)	30 mg kg^− 1^ (FAO 1983)
Zn	None	JECFA PMTDI 0.3–1.0 mg kg^− 1^ bw d^− 1^	0.3	None (repealed in 2018)	40–100 mg kg^− 1^ (FAO 1983)
Se	None	FAO/WHO RNI 26–34 µg day^− 1^; UL 400 µg day^− 1^; EFSA AI 70 µg day^− 1^	0.005	None	0.3–2 mg kg^− 1^ (source not traceable)
Mo	None	No JECFA value	0.005	None	None traceable
Microplastics	None	None	None	None	None – no limit exists

## Discussion

The present study provides evidence that braaied (open fire grilled) fish sold along South African roadways contains measurable levels of microplastics and metals, presenting potential implications for food safety and public health. The results collectively indicate that roadside–processed fish can accumulate multiple contaminant types, reflecting interactions between ecological contamination in aquatic habitats and possible contributions from handling, processing, and local environmental exposure. These findings align with the growing global concern regarding chemical and particulate contamination in small scale and informal food value chains (Smith et al. 2018). Microplastics and elements were detected in every fish sampled, and both varied significantly among stalls. Vendor behaviour was not quantified in this study, however, so the differences among stalls cannot be attributed to vending practice alone: they may equally reflect the environment in which the fish were produced or captured and contamination accrued elsewhere along the fish value chain before purchase.

The concentrations of the 12 elements recorded in braaied fish from roadside vendors varied among stalls, and mean Cr, Cu, Se and Mo concentrations exceeded internationally recommended guideline levels at some stalls. Elevated Cr and Fe may reflect exposure to urban atmospheric pollutants and/or degraded cooking surfaces, while Cu, Se and Mo may point to anthropogenic sources such as vehicle emissions, lubricants and metal rich dust; these interpretations are inferential, as no source apportionment was undertaken. The spatial pattern is nevertheless consistent with previous findings that atmospheric deposition of road–derived microplastics, vehicle exhaust particulates and resuspended dust can contaminate food prepared or sold outdoors (Chu et al. 2021; Premarathna et al. 2025). Vending conditions such as uncovered display, proximity to dusty roads and the use of exposed preparation surfaces (Appendix 1) may have contributed to the contamination observed, although this was not tested directly.

Across the nine sampled sites, microplastics were consistently detected in the edible muscle tissue, with mean concentrations exceeding values typically reported for fresh, unprocessed fish (see Jovanović 2017; Dalu et al. 2023, 2024). Microplastics may therefore be introduced both from the environment in which the fish were produced or captured and during capture, handling, storage and processing. Research (e.g., Li et al. 2022; Ma et al. 2025) has found that heat based cooking methods, including grilling and barbequing, can alter microplastic structure but do not completely remove them. For example, polymer particles fragment further, potentially increasing particle count. In this study, the predominance of fibres and films is consistent with trends observed in urban impacted fisheries (Naidoo et al. 2020; Dalu et al. 2023). Fibres are typically associated with laundry effluent and atmospheric fallout, while fragments correspond to environmental degradation of larger plastic materials (Dris et al. 2016; Acharya et al. 2021; Niu et al. 2024). Therefore, roadside vending environments, which often exhibit high dust and particulate loads, likely contribute to contamination during handling or display (Jaiswal et al. 2025). Equally, the elevated particle counts may reflect contamination of the source waters from which the fish were harvested, and the present design cannot separate the two contributions.

Polymer composition profiles were dominated by low density plastics associated with packaging, fishing and consumer goods, which are commonly recorded in urban and peri–urban environments (Parashar and Hait 2023; Jaiswal et al. 2025). This reinforces earlier work demonstrating that secondary microplastics derived from weathered waste materials are major contributors to environmental contamination near human activity centres (Andrady 2017). The observed polymer diversity also indicates multiple contamination pathways, including environmental uptake by fish prior to capture and secondary contamination during storage, handling, and braaing.

Although the toxicological implications of microplastic ingestion remain uncertain, the measured microplastic loads, combined with elevated concentrations of metals such as Cr and Se, indicate potential exposure of concern, particularly for children and other consumers of low body mass. The results are consistent with recent assessments showing that microplastic associated metals may pose combined risks when consumed through contaminated seafood (Bouwmeester et al. 2015; Revel et al. 2021). Intake estimates highlight repeated exposure for regular consumers. Using national per capita consumption values (Maulu et al. 2024), daily exposure from a typical 18.1 g fish portion results in appreciable particle intake, particularly in samples with high microplastic concentrations. When normalised per kilogram of body weight, exposure is highest for children and other individuals of low body mass, who therefore carry a disproportionately greater potential exposure burden (Smith et al. 2018; Al Mamun et al. 2023). Although the toxicological significance of ingested microplastics remains under active investigation, a growing body of evidence indicates that microplastics can act as vectors for chemical additives and adsorbed pollutants, including metals and persistent organic pollutants (Li et al. 2023).

Essential macro elements such as Ca, K, Mg and Na were abundant, consistent with natural physiological roles in fish. However, the significant among stall variation in Cr, Mn, Fe, Se and Mo (Table 1) indicates an environmental or handling influence on element loading. This variation is consistent with the hypothesis that pollution sources associated with roadsides, combustion activity, and informal waste disposal contribute to contamination in fish sold in these areas. Similar spatial patterns have been reported in fish exposed to polluted urban waterways or caught and handled in contaminated environments (Isangedighi and David 2019; Gulati et al. 2022).

Microplastics can adsorb metals on their surfaces and act as transport vectors, potentially increasing localised concentrations in biota (Holmes et al. 2012; Gao et al. 2019). Experimental evidence shows that metals such as Pb and Cd bind readily to oxidised polyethylene and polypropylene surfaces (Holmes et al. 2012; Gao et al. 2019), which were common polymer types identified in this study. No correlation between microplastic and element concentrations was tested at the stall level in the present dataset, so any co–variation between the two contaminant groups remains untested here. This raises the possibility that some proportion of the metal load measured in the edible tissue is associated with both ingested and environmental microplastics. Although the present study did not directly test metal microplastic binding interactions in fish tissues, the co–occurrence of high microplastic loads and elevated metal levels at specific sites warrants further investigation. Braaied fish sold by roadside vendors may also undergo contamination during preparation. Cooking over open fires, often using treated or waste wood, has been shown to release trace metals and combustion by–products that can deposit on food (Hasan et al. 2009). Additionally, storage on plastic trays, handling with synthetic materials, and exposure to roadside dust can further introduce or increase contaminant loads. These findings align with studies from Nigeria, Kenya, and Ghana that report elevated polycyclic aromatic hydrocarbons (PAHs), metals, and microplastics in street vended grilled fish (Ogwu and Izah 2025). The significant environmental influence on both microplastics and metals suggests that fish handling, storage practices, and ambient air quality contribute substantially to postharvest contamination. This aligns with growing evidence that food sold in open–air informal markets can accumulate microplastics from atmospheric fallout, contaminated contact surfaces, or dust settling during cooking and storage (Wang et al. 2021).

Element concentrations in fish muscle varied significantly among the nine stalls for Na, Cr, Mn, Fe, Se and Mo (Table 1), and mean Cr, Cu, Se and Mo concentrations approached or exceeded recommended international food safety limits at some stalls. Lead and cadmium were not among the elements measured in this study. The variation among sites, demonstrated statistically through one–way ANOVA, reflects spatial heterogeneity in pollution sources. Such heterogeneity is typical as it is influenced by industrial, agricultural, and urban effluents (Utete and Fregene 2020). The regression analyses using standard length and body weight revealed weak to moderate relationships between fish size and contaminant burdens. This pattern suggests that, while size related accumulation occurs, much of the particle and element load may be recent or externally derived rather than solely age or trophic position dependent; no bioaccumulation factors were calculated, so accumulation is inferred from the size relationships alone. This is particularly likely in systems experiencing acute or chronic contamination pulses.

Essential elements (i.e., Na, Mg, P, K and Ca) were detected at concentrations far above established baseline levels in freshwater fish, contradicting the assumption that these elements reflect solely natural biochemical composition. For example, Na levels exceeded typical fish values (50–500 mg kg^− 1^) by nearly two orders of magnitude. Similarly, Ca normally 1 000–5 000 mg kg^− 1^ reached over 60 000 mg kg^− 1^ in some samples. Such inflated concentrations suggest post–harvest contributions, including heavy salting prior to grilling, mineral–rich dust deposition along roadside stalls, and leaching from calcareous ash or metal cookware. These findings emphasize that essential elements can become artificially elevated in informal food environments. The main implication for consumers is nutritional as well as toxicological: sodium intakes of this magnitude are relevant to hypertension risk in a population in which dietary salt intake is already high, whereas the elevated Cr, Cu, Se and Mo concentrations are the more direct toxicological concern. For toxic and trace metals, exceedances of recommended limits were also observed, with elevated Cr levels aligning with contamination patterns in fish exposed to traffic emissions, industrial debris, and poorly managed waste (Boahen 2024). High Fe concentrations may originate from rusted grills or Fe–rich dust, while elevated Cu likely reflects contact with brass–or copper–based cooking equipment or atmospheric fallout (Usero et al. 2004). Selenium and Mo concentrations were also above expected freshwater fish ranges (EFSA 2014), raising concern because both elements have narrow thresholds between essentiality and toxicity.

No statutory maximum level in fish exists for any of the 12 elements measured here. Codex sets maximum levels in fish only for Pb, Cd, As and Hg (Codex Alimentarius Commission 1995), and South African regulation covers those four plus Hg and Sn, having repealed the Cu and Zn limits of R.500 of 2004 (Department of Health 2004, 2018). The benchmarks applied here are limits compiled by the FAO from national legislation then in force (FAO 1983), so an exceedance indicates a concentration unusual for edible fish tissue rather than a breach of a legal threshold. Against those benchmarks the trace element burdens were largely unremarkable: only Cr at site 1, iron at site 4 and Zn at site 2 approached or reached their screening values, and both Cr and Fe varied significantly among stalls (Table 1). Manganese exceeded its benchmark at every stall, but it is homeostatically regulated in vertebrates (US EPA 1998) and the 1983 value is weakly founded. This pattern of localised enrichment at particular stalls, rather than generalised contamination, points to stall-specific post-harvest sources such as degraded cooking surfaces or road dust (Hasan et al. 2009; Boahen 2024). The macro-elements are the more consequential finding, and their absence from food-safety instruments reflects their status as nutrients rather than their irrelevance to health. A single 100 g portion delivers 1.0–2.2 g of sodium, at or above the entire WHO daily limit for adults (WHO 2012), and calcium concentrations exceed the adult recommended nutrient intake several-fold per portion (FAO/WHO 2004). Heavy salting before grilling and ash or dust deposition during open-fire cooking are the plausible contributors (Hasan et al. 2009; Usero et al. 2004). For regular consumers of street-vended braaied fish the sodium load is the more immediate concern.

From a public health perspective, the levels of some contaminants detected in this study warrant attention. Chronic exposure to toxic metals is associated with renal dysfunction, developmental impairment, neurotoxicity and cardiovascular disease (Tchounwou et al. 2012); Cd and Pb were not measured here, but chronically elevated intakes of Cr, Cu, Se and Mo raise comparable, if less severe, concerns. While ingestion of microplastics has yet to be conclusively linked to specific human health outcomes, the existing literature raises concerns about inflammation, immune dysregulation, and chemical exposure from sorbed pollutants (Leslie and Depledge 2020). For communities that rely heavily on fish as a dietary protein source particularly low income households purchasing affordable street vended fish, the cumulative exposure could be appreciable. The findings of this study reinforce the need for targeted interventions to reduce contamination in both fish habitats and processing environments. Improving waste management, addressing upstream pollution sources, and implementing basic hygienic practices during roadside fish preparation could reduce contaminant loads. Moreover, public education on safer cooking methods and vendor training could help reduce exposure risks without undermining livelihoods that depend on informal fish marketing. Thus, this study contributes to the growing body of research highlighting the complexity of contamination pathways in small scale fisheries. The co–occurrence of microplastics and metals in braaied fish sold at roadside markets underscores the importance of holistic food safety assessments that account for both environmental exposure and post harvest contamination. Continued monitoring, coupled with improved regulatory oversight, is essential to safeguard consumer health while supporting sustainable and safe fish value chains in South Africa. Stalls situated near high traffic roads or waste impacted areas displayed higher mean concentrations, which is consistent with, but not proof of, an influence of airborne pollutants and surface contamination during handling and preparation. The results also reinforce the value of linking contamination to human dietary exposure, as consumers relying on informal vendors may be exposed repeatedly to elements at or above recommended limits. One important aspect would be to track fish from the farm to the consumer; tracing the supply chain can help identify key points of contamination.

This study has several limitations that qualify its conclusions: (i) the fish were purchased from vendors rather than sampled at their point of origin, and the provenance of individual fish could not be verified. Therefore, differences among stalls cannot be attributed to roadside vending conditions alone and may partly reflect the environments in which the fish were produced or captured; (ii) no uncooked or pre–display control fish were analysed, so the relative contributions of pre– and post harvest contamination could not be separated, and the post harvest pathway remains a plausible rather than a demonstrated explanation; (iii) vendor practices were not recorded with a structured instrument, so the environmental context of each stall is described qualitatively; (iv) with four fish per stall (*n* = 36) statistical power is limited, and non–significant results should not be read as evidence of homogeneity; (v) particle size classes were not quantified, no bioaccumulation factors were calculated, and microplastic element correlations were not tested at the stall level; and (v) exposure is expressed as particle and element intake rather than as estimated daily intakes, target hazard quotients or hazard indices, because oral reference doses are not available for microplastic particles or for all of the elements measured, thus the estimates presented here should therefore be read as screening level indicators rather than as a formal health risk assessment.

## Conclusion

Microplastics and a range of elements were present in the edible muscle of braaied fish sold by informal roadside vendors in Thohoyandou, and both varied significantly among stalls. Fibres and films dominated the particles recovered, and polyethylene, polypropylene, polystyrene and polyvinyl chloride accounted for most of the polymers identified. Mean Cr, Cu, Se and Mo concentrations exceeded recommended guideline values at some stalls, and estimated intakes of ~ 180–790 particles per meal indicate that consumers of street vended fish are exposed to microplastics at every stall sampled. Because the fish were sampled at the point of sale only, this study cannot apportion contamination between the source environment and post harvest handling; tracing fish along the value chain, from production to consumer, is the necessary next step. In the interim, improved waste management, covered display and cleaner preparation surfaces are low cost measures that could reduce contaminant loads without undermining the livelihoods that depend on informal fish marketing.

## Supplementary Information

Below is the link to the electronic supplementary material.

Supplementary Material 1

## Data Availability

All data have been presented in the article.

## Declarations

All authors have read, understood, and have complied as applicable with the statement on “Ethical responsibilities of Authors” as found in the Instructions for Authors.
