# Supplementary material for: Assessment of Microplastic and Element Contamination in Fish Sold by Informal Roadside Vendors in Limpopo Province, South Africa
Source: Arch Environ Contam Toxicol. 2026 Oct 26;91(3):28. doi: 10.1007/s00244-026-01223-3 (PMC13710681; doi:10.1007/s00244-026-01223-3)
Supplement: Supplementary file 1 [file 244_2026_1223_MOESM1_ESM.docx]

**Figure S1.** Various braai or barbecue fish selling areas along the roads of the Thulamela Municipality area
